# A comparison of social attitudes, professional and institutional identities and acculturative stress between podiatry and other health professional students

**DOI:** 10.1186/1757-1146-4-S1-P16

**Published:** 2011-05-20

**Authors:** Verona du Toit, Andrea Bialocerkowski, Roslyn Weaver, Rosalind Bye, Yenna Salamonson

**Affiliations:** 1 School of Biomedical and Health Sciences, University of Western Sydney, Campbelltown Campus, Sydney, NSW 2560, Australia; 2 Family and Community Health Research Group, School of Nursing and Midwifery, University of Western Sydney, Campbelltown Campus, Sydney, NSW 2560, Australia

## Background

In multicultural societies, such as Australia, it is important for health professional students to possess skills to interact positively with people from a range of cultures. This study describes first-year podiatry students’ social attitudes, professional and institutional identities and acculturative stress; and compares these with other health professional students in the school.

## Methods

Thirty-three out of forty-six enrolled first-year podiatry students completed surveys at a large, culturally diverse university in Sydney, Australia. Demographic data and standardised measures of English language acculturation, acculturative stress, universe diverse orientation and professional and institutional identities were collected. Surveys were also administered to first-year physiotherapy, occupational therapy, nursing and medical students at the same university. Data were entered into and analysed in SPSS version 18. Descriptive statistics and Kruskal-Wallis tests were used to describe and compare the student cohorts.

## Results

Seventy-three percent of first-year podiatry students were born in Australia although 36% of these students speak a language other than English at home. Podiatry was the first course preference for 73% of students, and 34% of the cohort reported having a close friend in the same course. 64% were in paid employment at the time of the study, and of these 71% worked in a non-health-related area.

## Conclusions

Trends were identified which differentiated the health professions. When compared with other health professional students, podiatry students had relatively low levels of acculturative stress and moderate levels of professional and institutional identity. This suggests that first-year podiatry students (after 12 weeks of study) have appropriate attitudes that will facilitate the development of cultural competence with further study. The data to date has shown similar trends for physiotherapy and occupational therapy students in the School of Biomedical and Health Sciences.

